# Editorial

**DOI:** 10.1120/jacmp.v6i2.2136

**Published:** 2005-05-21

This issue, I would like to present a guest editorial from my colleague at *Medical Physics*, Dr. William R. Hendee. Bill graciously responded to my invitation to discuss the maintenance of certification issue, which I believe is the most important issue facing our profession. Thanks, Bill; we all appreciate what you have to say.

Michael D. Mills PhD

## Performance and accountability in medical physics: A point of view

William R. Hendee PhD

In the United States, we seem to be enamored by the idea that professionals should perform as expected and that they should be accountable for their performance by verification against measurable and quantifiable standards. In part, this idea has grown out of the so‐called decline of deference in which the veneer of invincibility of teachers, ministers, doctors, auditors, and other professionals in our society has eroded over the past few decades. It is also a reaction to recent revelations of questionable ethical values and behaviors of business, industry, religious, and political leaders. But underneath these reactions lies a fundamental truth: professionals should perform as they are educated and trained to function, and they should be held accountable for their performance because, fundamentally, they are entrusted with the public's welfare.

Certainly professional performance and accountability are essential in medicine; no field surpasses health care in the depth and breadth of its impact on the lives and well‐being of people. Good performance in health care can save lives and reduce suffering—and poor performance can cost lives and increase suffering. Health‐care professionals are entrusted by patients and families with the expectation of good performance, and health‐care professionals owe patients and families their willingness to be transparent, ethical, and accountable in the application of their skills and knowledge to the patients' benefit. This social contract between those who provide care and those who receive it is beyond question. What remains questionable, however, is how to document performance and how to assure accountability. These questions are pertinent to every field of health care, including medical physics.

Licensure by a quasi‐governmental agency has traditionally been one approach to verification of the credentials of health‐care professionals. This approach suggests that the individuals should be able to perform competently in their roles within a health‐care setting. Only four states (Texas, Florida, Hawaii, and New York) license medical physicists. In most states, medical physics licensure does not exist because there has been insufficient interest to mount the lobbying effort required to move a licensing bill through the state legislature. Licensure does not ensure that medical physicists perform their duties as expected, and it does little for accountability of medical physicists to the public that they serve. But it does set a threshold for the credentials of medical physicists that serves to weed out those whose qualifications fall short of what is considered essential.

Certification by a process established by peers is a second and more widespread approach to documentation that professionals possess the knowledge and experience to practice in a discipline. Medical physicists can be certified in diagnostic radiological physics, therapeutic radiological physics, and medical nuclear physics by the American Board of Radiology professional duties. Certification merely documents that individuals at one particular point in time possessed the knowledge and experience considered necessary to practice a particular profession.

Accreditation is another mechanism occasionally referenced as a way to ensure that the performance and accountability of professionals meet accepted standards. This interpretation is misguided because accreditation does not apply to individuals; it applies to programs of education and training; that is, professionals can graduate from accredited educational programs, but they cannot become accredited as individuals. In medical physics, accreditation of graduate and residency education and training programs is provided by the Commission on Accreditation of Medical Physics Educational Programs (www.campep.org), an organization sponsored by the American Association of Physicists in Medicine, American College of Medical Physics, American College of Radiology, and the Canadian College of Physicists in Medicine. Accreditation ensures that a graduate or residency program in medical physics satisfies certain peer‐determined standards for educational content and clinical experience. It provides no direct assurance that individuals graduating from accredited programs will perform competently and with accountability to the public.

So even with licensure, certification, and accreditation, the questions of how to document performance and how to assure accountability in medical physics remain unanswered. Yet, medical physicists, like other health‐care professionals, are expected to answer these questions. This expectation reflects in part society's demand for heightened accountability of health‐care professionals, and in part a self‐imposed desire of health‐care professionals to measure up to a high level of accountability over their course of practice. An effort to meet this expectation has yielded the evolving process of maintenance of certification (MOC) now confronting medical physicists (and radiologists and radiation oncologists) who have time‐limited certificates issued by the American Board of Radiology (ABR). Physicists with lifetime certificates from the ABR are also encouraged to engage in the MOC process as a testament to their dedication to professional performance and accountability. The MOC process is designed to document that medical physicists perform their professional duties at a level deemed acceptable to their peers, and to provide greater transparency in the performance of those duties in an effort to be more accountable to patients, families, and the public in general.

Maintenance of ABR certification has four components:

- professional standing
- lifelong learning and self‐assessment
- cognitive expertise, and
- assessment of performance in practice

For each of these components, six competencies should be demonstrated:

- medical knowledge
- patient care
- interpersonal and communication skills
- professionalism
- practice‐based learning and improvement, and
- systems‐based practice

For medical physicists, professional standing will be demonstrated by letters of attestation of performance, licensure and regulatory agency certification, and expertise‐based appointments or recognition; lifelong learning and self‐assessment will be assessed by continuing education credits and self‐directed education projects; cognitive expertise through periodic written examinations; and assessment of performance in practice by documentation of specific functions and responsibilities carried out in practice. These requirements are described in greater detail on the ABR's web site.

Medical physicists are experts who perform their professional responsibilities with diligence and dedication, and who are actively engaged in continuing education demanded by their involvement in a rapidly evolving specialty. They are all busy people, and insertion of the MOC process into their already complex schedules makes their lives even more complicated. But MOC is happening, and it needs to happen so that physicists can demonstrate performance at a level consistent with their education and training, and accountability for that performance in the service of patients, families, and the public.

Journal of Applied Clinical Medical Physics

**Table 1:** 

**Editorial Board 2005**	
Editor‐in‐Chief	Michael D. Mills
Deputy Editor‐in‐Chief	Timothy D. Solberg
Associate Editor‐at‐Large	Richard Stark
**Associate Editors**	
Radiation Oncology Physics	Nzhde Agazaryan, B. Gino Fallone,
	John Gibbons, Michael Herman,
	Edwin McCullough, Janelle Molloy,
	Matthew Podgorsak, Nikos Papanikolaou,
	Mehrdad Sarfaraz, Lu Wang
Medical Imaging	Walter Huda, William Pavlicek
Radiation Measurements	Larry DeWerd
Radiation Protection & Regulations	James Deye
Nonionizing Topics	Bhudatt Paliwal
Other Topics	Timothy Solberg
Book/Media Reviews	James Smathers
Editorials	John Horton
**Journal Production Team**	
Manuscript Manager	Patricia Walker
Copy Editor	Betty R. Robinson
Layout Editor	Melanie Marques
Proofreader	Heather Shand

